# Systematic Myostatin Expression Screening Platform for Identification and Evaluation of Myogenesis-Related Phytogenic in Pigs

**DOI:** 10.3390/bioengineering10101113

**Published:** 2023-09-22

**Authors:** Bor-Rung Ou, Ming-Hua Hsu, Ling-Ya Haung, Chuan-Ju Lin, Li-Li Kuo, Yu-Ting Tsai, Yu-Chia Chang, Wen-Yuh Lin, Tsung-Chien Huang, Yun-Chu Wu, Jan-Ying Yeh, Yu-Chuan Liang

**Affiliations:** 1 Department of Animal Science and Biotechnology, Tunghai University, Taichung 407, Taiwan; brou@thu.edu.tw (B.-R.O.); niyamurry@gmail.com (L.-Y.H.); edhuang68@gmail.com (T.-C.H.);; 2 Department of Chemistry, National Changhua University of Education, Changhua 500, Taiwan; minghuahsu@cc.ncue.edu.tw; 3 Agricultural Biotechnology Research Center, Academia Sinica, Taipei 115, Taiwan; myd208@gate.sinica.edu.tw (C.-J.L.);; 4 Department of Food Nutrition and Health Biotechnology, Asia University, Taichung 413, Taiwan; 5 College of Agriculture and Health, Tunghai University, Taichung 407, Taiwan

**Keywords:** myogenesis, skeletal muscle, myostatin, *Glycyrrhiza uralensis*, feed additive

## Abstract

Skeletal muscle growth in livestock impacts meat quantity and quality. Concerns arise because certain feed additives, like beta-agonists, may affect food safety. Skeletal muscle is a specialized tissue consisting of nondividing and multinucleated muscle fibers. Myostatin (MSTN), a protein specific to skeletal muscle, is secreted and functions as a negative regulator of muscle mass by inhibiting the proliferation and differentiation of myoblasts. To enhance livestock muscle growth, phytogenic feed additives could be an alternative as they inhibit MSTN activity. The objective of this study was to establish a systematic screening platform using MSTN activity to evaluate phytogenics, providing scientific evidence of their assessment and potency. In this study, we established a screening platform to monitor myostatin promoter activity in rat L8 myoblasts. Extract of *Glycyrrhiza uralensis* (GUE), an oriental herbal medicine, was identified through this screening platform, and the active fractions of GUE were identified using a process-scale liquid column chromatography system. For in vivo study, GUE as a feed additive was investigated in growth-finishing pigs. The results showed that GUE significantly increased body weight, carcass weight, and lean content in pigs. Microbiota analysis indicated that GUE did not affect the composition of gut microbiota in pigs. In summary, this established rodent myoblast screening platform was used to identify a myogenesis-related phytogenic, GUE, and further demonstrated that the active fractions and compounds inhibited MSTN expression. These findings suggest a novel application for GUE in growth performance enhancement through modulation of MSTN expression. Moreover, this well-established screening platform holds significant potential for identifying and assessing a diverse range of phytogenics that contribute to the process of myogenesis.

## 1. Introduction

Skeletal muscle is the largest tissue and represents approximately 40% of total body mass, and it is a multifunction tissue that plays a fundamental role in regular daily activities, including not only motor function and respiration, but also energy storage within the body in the form of proteins [1,2]. Furthermore, skeletal muscle represents the primary source of animal proteins in economically significant animals destined for human consumption. The growth and maturation of skeletal muscle has a direct impact on meat production [3]. Various feed additives, such as beta-agonists and anabolic steroids, are used in livestock to directly promote muscle growth [4]. Beta-agonists increase skeletal muscle mass and decrease fat mass in animals by stimulating muscle protein synthesis [5]. In addition, recombinant bovine somatotropin enhances growth rates, feed conversion, and lean meat in beef cattle [6]. However, it is crucial to consider the disadvantage and food safety of these feed additives. Beta-agonist residues in animal meats and organs raise food safety concerns [7], and some beta-agonists also generate risks of tachycardia, hypertension, and cardiac hypertrophy, potentially leading to heart failure in animals and humans [8]. On the other hand, anabolic steroids cause endocrine disruptions and are reported to be associated with carcinogenic, immunotoxic, mutagenic, and teratogenic effects, leading to irreversible consequences [9,10]. Hence, searching for feed additives that not only are safe but also directly promote muscle growth is of utmost importance.

Phytogenic additives are derived from various plant parts, such as leaves, roots, seeds, flowers, buds, or bark, and their extracts. Some phytochemical compounds have a long history of use in human medicine and are known for their pharmacological effects [11,12,13]. Owing to their natural and safe properties, phytogenic feed additives become more popular as natural alternatives than conventional additives, such as antibiotics and growth-promoting hormones [13,14,15]. The majority of phytogenic feed additives are recognized for their diverse biological activities in antimicrobial, antioxidant, anti-inflammatory, gut health, and digestive-enhancing properties to promote growth performances indirectly [16,17,18]. However, only a limited number of phytogenic feed additives are addressed to promote skeletal muscle growth. To improve the growth performance of animals or livestock, a systematic rodent myoblast screening platform that targeted myostatin (MSTN) promoter activity was established to identify several protentional phytogenics involving myogenesis enhancement.

MSTN is a secreted protein that serves as an inhibitory factor in the development of skeletal muscle [19,20]. This gene is remarkably conserved across mammals, and mutations resulting in its loss of function have been documented in diverse species, such as cattle, sheep, pigs, rabbits, and humans. These mutations result in increased skeletal muscle mass and the emergence of a “double-muscle” phenotype [21,22,23]. Therefore, inhibition of *MSTN* gene expression should potentially enhance skeletal muscle development in both muscle cells and animals.

To enhance skeletal muscle growth of livestock, phytogenic feed additive may be a potential alternative solution to inhibit NSTN activity in animals. The goal of this study was to establish a systematic screening platform for evaluating the activity of phytogenics through MSTN activity, aiming to offer scientific evidence regarding their effectiveness and potency.

## 2. Materials and Methods

### 2.1. Materials

We procured dried *Glycyrrhiza uralensis* and *Platycodon grandiflorus* roots, as well as dried plants without roots, from *Andrographis paniculata*, *Centella asiatica*, *Gynostemma pentaphyllum*, *Polygonum chinense*, *Portulaca oleracea*, *Saururus chinensis*, *Smilax china*, and *Taraxacum campylodes* from a reputable Chinese medicinal herb store in Taiwan (July 2020). Taq DNA polymerase was obtained from Kapa Biosystems (Roche, Basel, Switzerland). pMetLuc2 plasmid DNA and Ready-To-Glow™ Secreted Luciferase Reporter System were obtained from Clontech (Mountain View, CA, USA). Bovine serum albumin (BSA), T4 DNA ligase, and restriction enzymes (BglII and BamHI) were purchased from Promega (Madison, WI, USA). Gancaonin G and licoisoflavone A were obtained from ChemFaces (Wuhan, Hubei, China). Cudraflavone-C was purified in Dr. Liang’s laboratory (Figure S1). Methylthiazoletetrazolium (MTT), dimethyl sulfoxide (DMSO) and Geneticin™ selective antibiotic (G418 sulfate) were obtained from Sigma (St Louis, MO, USA). Dulbecco’s Modified Eagle Medium (DMEM) and fetal bovine serum (FBS) were obtained from Hyclone (Logan, UT, USA). Ethanol was obtained from J. T. Baker (Phillipsburg, NJ, USA). In this study, all other chemicals and solvents utilized were of reagent or high-performance liquid chromatography (HPLC) quality.

### 2.2. Plant Extract Preparation and Cells Transfection

The dried herbal plant material was ground into powder (20 Mesh), then mixed with 10 parts of 95% ethanol and stirred for 24 h. The liquid extract was filtered through a sieve (200 Mesh) and subsequently concentrated using a rotary vacuum evaporator. The crude herbal extracts were used for in vitro tests or fractionated to different fractions later. The Rat L8 myoblast cell line was obtained from American Type Culture Collection (Manassas, VA, USA), maintained in DMEM supplemented with 10% FBS, penicillin (100 U/mL), streptomycin (100 μg/mL), sodium pyruvate (1 mM), and glutamate (292 μg/mL) and incubated at 37 °C under 5% CO_2_. The 5′-flanking region (2.46-kb) upstream of the translation start site of the MSTN gene (GenBank accession no. AY204900) was amplified from mouse genomic DNA [24] by polymerase chain reaction (PCR) with a pair of specific primers containing *Bgl*II and *Bam*HI sites (Figure S2), respectively. The pMetLuc2-MSTN (Figure S2) reporter plasmid was constructed by inserting this 2.46-kb MSTN promoter into pMetLuc2 plasmid. The pMetLuc2-MSTN L8 cells (L8 MSTN-Luc cells) used in this study were derived by stable transfection of pMetLuc2-MSTN and selected with G418.

### 2.3. MTT Assay

For MTT assay, stable transfected L8 MSTN-Luc cells were counted with a hemocytometer (Hausser Scientific, PA, USA), and 2 × 10^4^ cells were seeded to each well on a 96-well plate in DMEM containing 5% FBS. The experiment was performed when the cells reached 80% confluence. Various concentrations of herbal extracts were used to treat the cells for 24 h at 37 °C, and then the cell proliferation was determined by MTT colorimetric assay [25]. Briefly, MTT reagent (2 mg/mL) was added to each well and incubated for 4 h at 37 °C. After adding dimethyl sulfoxide (DMSO) to each well, the OD_595_ of each well was measured by BioTek Powerwave XS microplate reader (Winooski, VT, USA).

### 2.4. Luciferase Assays

For luciferase activity, 2 × 10^4^ cells of stable L8 MSTN-Luc cells were seeded onto 96-well plates with DMEM containing 5% FBS. Secreted Metridia luciferase activity was measured using the Ready-To-Glow™ Secreted Luciferase Reporter Systems after 24-h treatment of various herbal extracts. The culture medium was removed to mix with luciferase substrate and VICTOR3 multilabel counter (PerkinElmer, Turku, Finland) was used to measure luciferase activity. The luciferase activity was normalized with cell proliferation prior to statistical analysis.

### 2.5. Reverse Transcription-Polymerase Chain Reaction (RT-PCR)

Total RNA was extracted from L8 cells using the Trizol Reagent (Invitrogen, Carlsbad, CA, USA) according to the manufacturer’s instructions, then reverse transcribed into complementary DNA using Revert Aid Reverse Transcriptase (Thermo Fisher Scientific, Waltham, MA, USA) and oligo(dT) as primer. The PCR was performed by Taq DNA polymerase using primers specific to MSTN (5′-CCAGGCACTGGTATTTGGCA-3′ and 5′-AAGTCTCTCCGGGACCTCTT-3′). Amplification was conducted using a GeneAmp PCR System 9700 (Applied Biosystems, Foster City, CA, USA) with the following conditions: an initial cycle at 94 °C for 5 min, followed by 35 cycles at 94 °C for 30 s, 55 °C for 30 s, and 72 °C for 30 s. The cycling concluded with a final elongation step at 72 °C for 5 min. The β-actin was used as the internal control. Amplicons after 2% agarose gel electrophoresis were visualized by a Doc Print System (Vilber Lourmat, Marne-la-Vallée, France), and the relative band intensities were assessed using the ImageJ software (NIH, Bethesda, MD, USA).

### 2.6. GUE Preparation

The grinded *G*. *uralensis* (1 kg) was extracted using 95% ethanol solution for 24 h prior to evaporation. After evaporation, the extract was applied to a process-scale liquid column chromatography system (Isolera Flash Puri, Biotage, Uppsala, Sweden). The separation was performed using a C18 column (KP-C18-HS column, Biotage), and the solvent gradient was 95% EtOH in H_2_O, and the extract was separated into 10 fractions under UV 245 nm. The major components of G9 were purified and isolated by using semi-preparative HPLC (Agilent, Palo Alto, CA, USA) on a RP-18 column (250 × 10 mm, Phenomenex, Torrance, CA, USA) at a flow rate of 1.0 mL/min, detected at UV 254 nm, 280  nm, and 300  nm. The chemical structures of the three compounds were determined through spectroscopic analysis. Electrospray ionization mass spectrometry data were acquired using an LCQ Advantage mass spectrometer (Thermo Finnigan, San Jose, CA, USA), while nuclear magnetic resonance spectra were recorded using Avance 500 and 300 MHz FT nuclear magnetic resonance spectrometers (Bruker, Bremen, Germany) at 500 MHz (^1^H) and 75 MHz (^13^C). These three major compounds of G9 were identified as gancaonin G, cudraflavone C, and licoisoflavone A by comparing their NMR, MS data, and optical rotations with those reported previously [26,27,28].

### 2.7. Animal Study

To investigate the beneficial effect of the GUE as a feed additive, the growth-finishing pigs were fed a standard diet or the diet containing GUE in the experimental ranch at Tunghai University (Taichung, Taiwan). Twenty barrows and twenty gilts ([Landrace × Yorkshire] × Duroc) with an initial average body weight (BW) of 12.24 ± 1.42 kg were randomly distributed among four treatment groups (control, low, medium and high concentrations with 10 pigs each group). All animals were monitored according to the previous standard procedure [29] and were acclimatized with the standard diet (Fwusow Industry Co., Ltd., Taichung, Taiwan) and free access to water for one week prior to the experiment. Mortality, body weight, feed and water intake were recorded weekly. At the end of experiment, hot carcass weights were recorded and used to calculate the carcass percentage. The length of each carcass spanning from the posterior edge of the symphysis pubis to the anterior edge of the first rib was measured, and the skin, bone, legs, shoulders, loins, bellies, tenderloins, backfat, and neck fat were collected using the simplified EC-reference method [30]. All experimental protocols received approval from the Institutional Animal Care and Use Committees at Tunghai University (Protocol no. 107-38).

### 2.8. Pyrosequencing and Data Analysis

The gut bacterial DNA extracted from pig feces in each treatment group at the end of the experiment were used as templates for PCR amplification with 16S rRNA primers. The 16S rRNA amplicons for the illumine MiSeq platform (Illumina, San Diego, CA, USA). In order to prepare the library, the primer pair sequences target the V3 and V4 regions, which generate a single amplicon of approximately 460 base pairs. Perform a limited cycle PCR to amplify the V3 and V4 region and incorporate Illumina sequencing adapters along with dual-index barcodes into the target amplicon. With the complete set of Nextera XT indices, it is possible to combine and sequence up to 96 libraries concurrently. Employing the MiSeq platform with paired 300-base pair reads and MiSeq v3 reagents, the ends of each read are merged to produce high-quality, full-length sequences of the V3 and V4 region within a single 65-h run. The results of 16S rRNA gene amplicons sequencing will be analyzed by MiSeq Reporter (Illumina), BaseSpace (Illumina), and Greengenes database (Lawrence Berkeley National Laboratory, Berkeley, CA, USA) to complete taxonomy and classification of microbiota GUE-treated or non-treated in pigs.

### 2.9. Statistics

The data is displayed as the mean ± standard error of the mean (SEM) and assessed for equal variance and normal distribution prior to statistical analysis using analysis of variance (ANOVA). A significance level of 0.05 was adopted. Fisher’s least significant difference (LSD) test was used for group comparisons.

## 3. Results

### 3.1. Establishment of Systematic Rodent Myoblast Screen Platform and Phytogenic Screening

**Figure 1:**
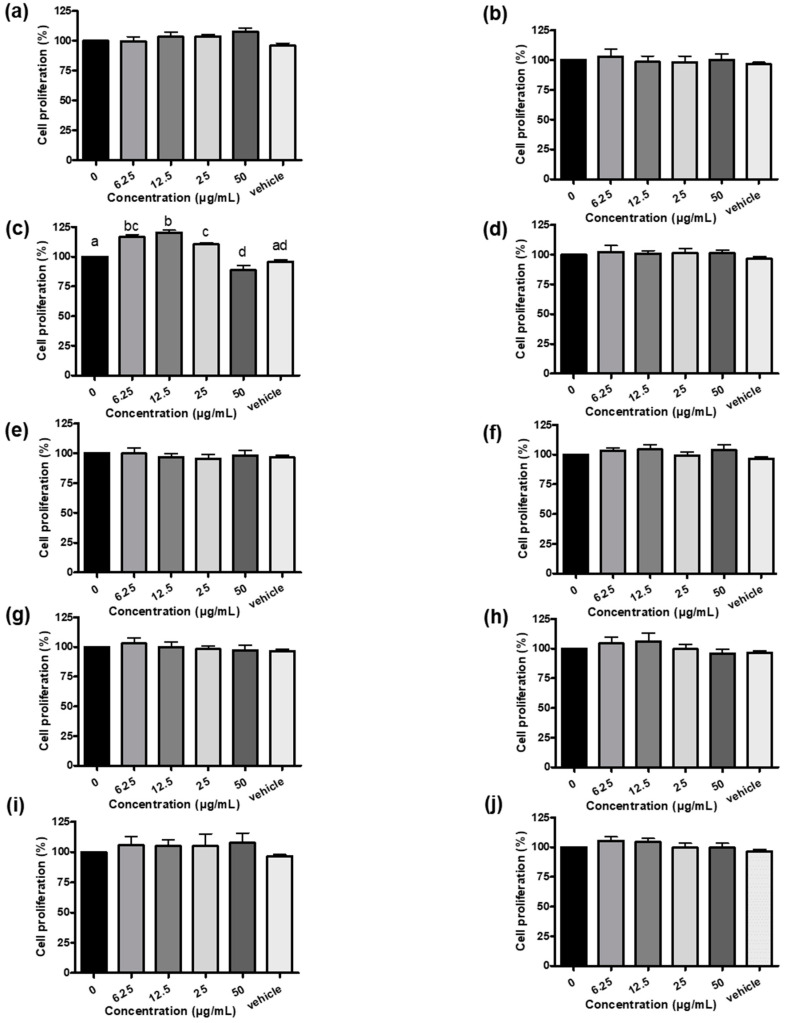
Effect of various herb extracts (**a**–**j**) on L8 MSTN-Luc cells proliferation. L8 MSTN-Luc cells were treated with various herbal extracts, *Andrographis paniculate* (**a**), *Centella asiatica* (**b**), *Glycyrrhiza uralensis* (**c**), *Gynostemma pentaphyllum* (**d**), *Platycodon grandifloras* (**e**), *Polygonum chinense* (**f**), *Portulaca oleracea* (**g**), *Saururus chinensis* (**h**), *Smilax china* (**i**) and *Taraxacum campylodes* (**j**), in different concentrations or with 1% ethanol as vehicle control. L8 MSTN-Luc cell proliferation was measured using MTT assay. Different letters (a–d) denote significant differences (*p* < 0.05) among treatments. Each data point represents the mean ± SEM derived from three separate and independent experiments.

**Figure 2:**
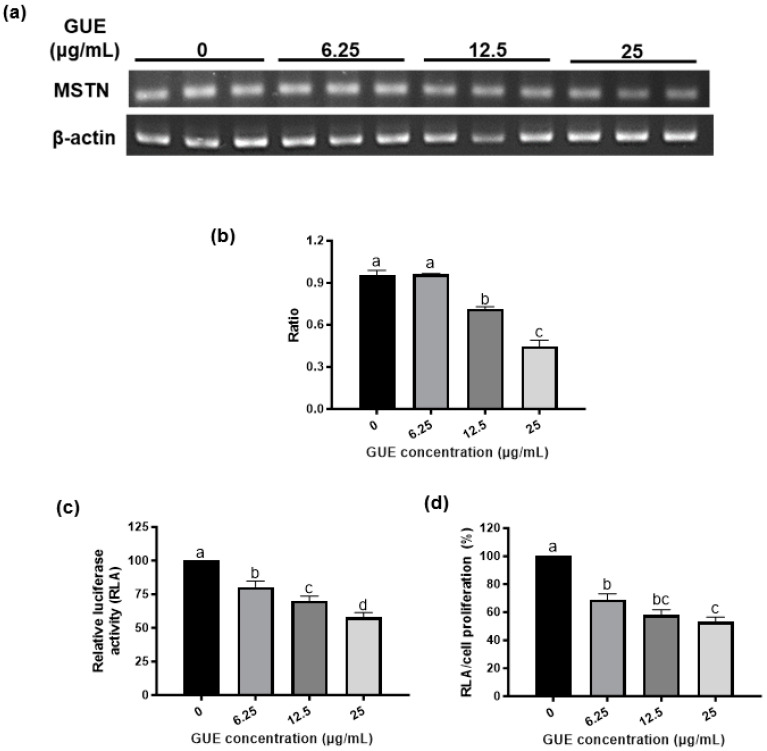
Effect of G. uralensis extract (GUE) on MSTN mRNA level and MSTN promoter activity in L8 MSTN-Luc cells. The effect of GUE on MSTN mRNA level were determined by reverse transcription-polymerase chain reaction (RT-PCR) (**a**). The relative ratio of MSTN mRNA level to internal control was calculated (**b**). The effect of GUE on cell proliferation was investigated by MTT assay (Figure 1c). MSTN promoter activity was measured by luciferase activity assay (**c**). The ratio of luciferase activity to cell proliferation (**d**) was used to evaluate GUE inhibitory effect. Different letters (a–d) denote significant differences (*p* < 0.05) among treatments. Each data point represents the mean ± SEM derived from three separate and independent experiments.

In this study, the myostatin-luciferase (MSTN-Luc) reporter gene was transfected into L8 cells successfully, and the stable clone (L8 MSTN-Luc cells) showed induced MSTN-Luc expression by dexamethasone at the effective concentrations reported previously [31,32] (Figure S3). Thus, the MSTN-Luc activity in L8 MSTN-Luc cells can be used as a cell-based assay for MSTN expression in myoblast to detect the bioactivity of phytogenics, plant extracts, fractions, or phytochemicals. The common herbal plants related to skeletal muscle diseases or regulation, *A*. *paniculate*, *C*. *asiatica* [33], *G*. *uralensis* [34], *G*. *pentaphyllum* [35], *P*. *grandifloras* [36], *P*. *chinense* [37], *P*. *oleracea* [38], *S*. *chinensis* [39], *S*. *china* [40] and *T*. *campylodes* [41], were selected to be screened in this study. Most herbal extracts, other than GUE, did not increase L8 MSTN-Luc cell proliferation (Figure 1c). The low-concentration GUE was found to have large significant effects (*p* < 0.05) on L8 MSTN-Luc cell proliferation, about 15% to 20% increase (Figure 1c). Similar patterns were observed from the luciferase activity results of L8 MSTN-Luc cells treated with various herbal extracts (Figure S3). The MSTN mRNA level in L8 MSTN-Luc cells treated with GUE were analyzed by RT-PCR. The decreased mRNA level of MSTN in the GUE-treated groups was concentration-dependent. The higher concentration of GUE led to greater inhibition (*p* < 0.05) of MSTN mRNA level in L8 MSTN-Luc cells (Figure 2a,b). Therefore, GUE with increased cell proliferation and luciferase inhibition (Figure 2c,d) was used for further in vitro studies.

### 3.2. Characterization and Identification of Active Fractions in GUE

**Figure 3:**
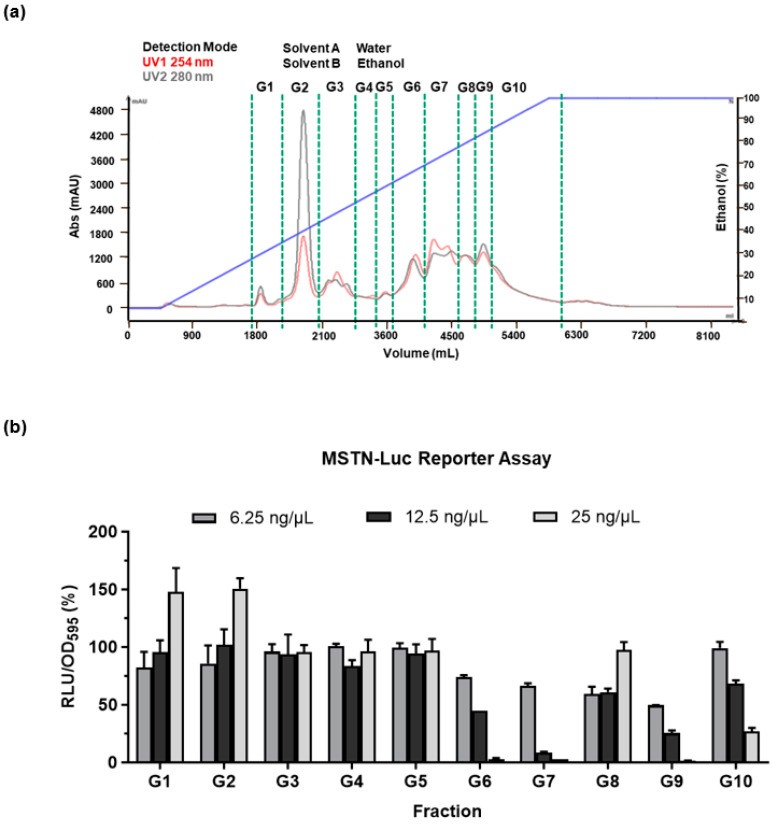
Systematic analysis of major active fractions of *G. uralensis* extract. The ethanolic extract of *G. uralensis* was purified using process-scale liquid column chromatography and separated into 10 fractions (**a**). The bioactive fraction was identified by the MSTN gene reporter assay in L8 MSTN-Luc cells treated with various GUE concentrations (**b**). The results are indicated as the ratio of luciferase activity to cell proliferation.

**Figure 4:**
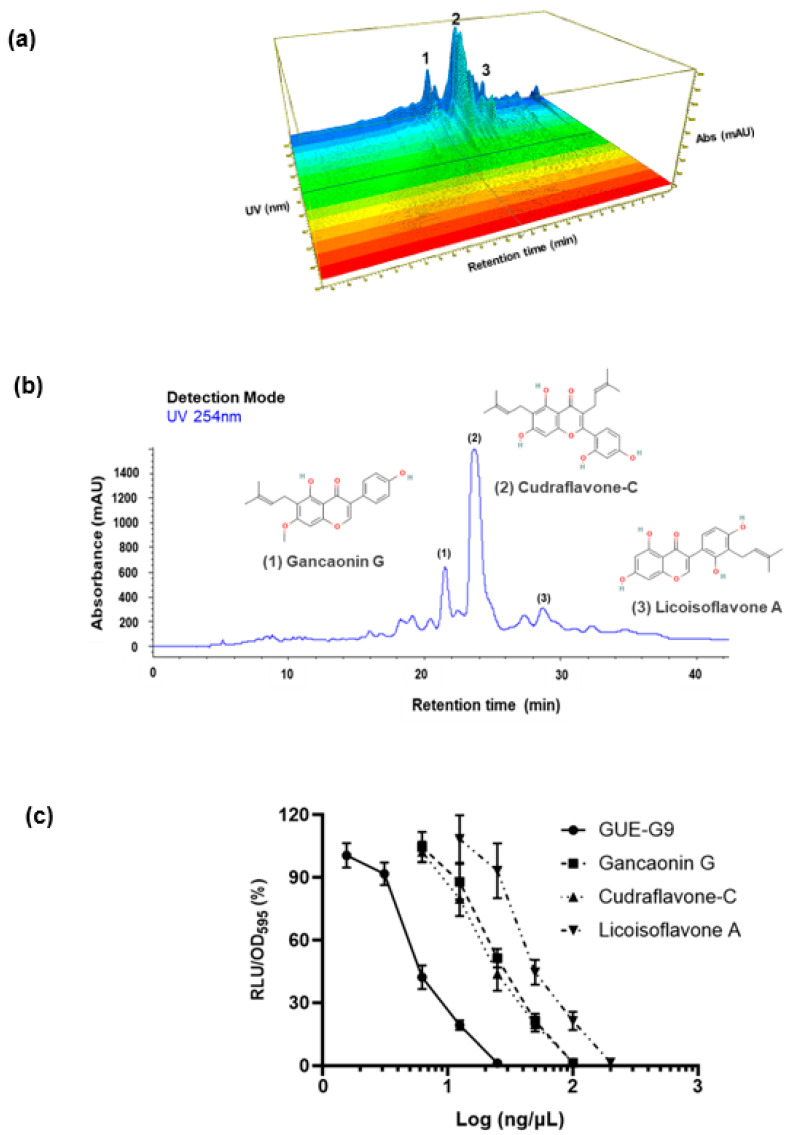
Chemical profile of the MSTN-inhibitory fraction (G9) in *G. uralensis* extract. The chemical profile of *G. uralensis* active fraction (G9) was analyzed using the RP-18 column and detected with DAD detection at 210–540 nm (**a**). Peaks of bioactive compounds and resulting chemical structures were identified as shown (**b**). Dose-response curves of three individual phytocompounds and the G9 fraction for MSTN suppression activity (**c**). Each data point represents the mean ± SEM.

The complex chemical composition of phytogenics makes it challenging to identify active compounds and mention batch-to-batch consistency. In this study, the process-scale liquid column chromatography was used to enrich biological activity of the GUE. The full-spectrum GUE was separated into 10 fractions (G1–G10) in a gradient elution mode (Figure 3a), and the activity of each fraction was examined by MSTN-reporter assay in L8 MSTN-Luc cells (Figure 3b). The G9 fraction was used for further purification and identification among the active fractions. Next, the G9 fraction was further purified by column chromatography using an RP-18 column (Figure 4a). Three bioactive phytocompounds were found in G9 fraction. Chemical structures of the bioactive compounds were then determined by nuclear magnetic resonance and mass spectrometry. As marked in the HPLC chromatogram, elution peaks that contained bioactive compounds and their respective chemical structures were identified as gancaonin G, cudraflavone-C, and licoisoflavone A (Figure 4b). The efficacy and potency of each bioactive compound for MSTN suppression as determined by L8 MSTN-Luc cell-based reporter assay was concentration-dependent (Figure 4c). The relative concentration of the maximal inhibition was GUE-G9 < gancaonin G < cudraflavone-C < licoisoflavone A, and the inhibitory trends were almost the same in each treatment group.

### 3.3. Effect of GUE as a Feed Additive on Growth Performance in Growth-Finishing Pigs

**Figure 5:**
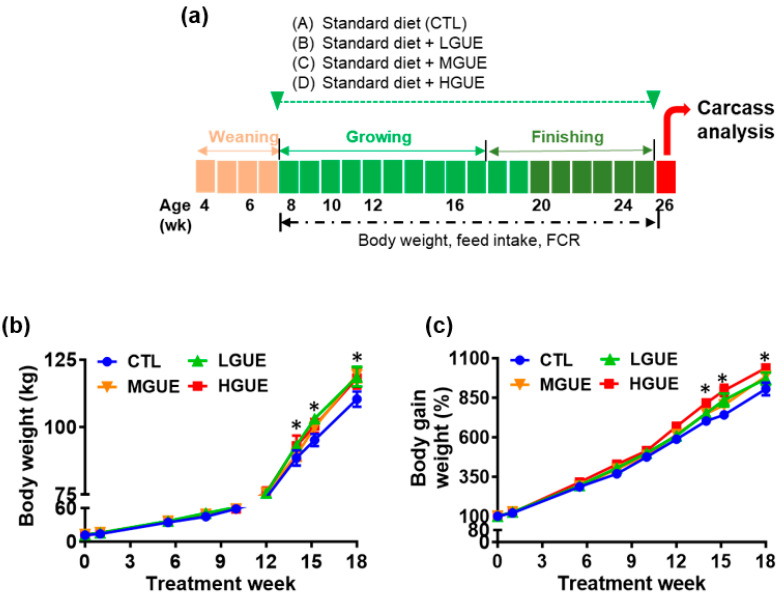
Effect of GUE as a feed additive on body weight in growth-finishing pigs. Flow charts show the timeline for evaluating of GUE effect as a feed additive on growth-finishing pigs (**a**). After weaning, pigs were randomized into four groups. Standard control diet (CTL), low GUE concentration (LGUE), medium GUE concentration (MGUE), and high GUE concentration (HGUE) treatment of 10 animals in each group. GUE was used as a feed additive for 18 weeks. Body weight (**b**) and body gain weight (**c**) in each treatment group was monitored before and after 18-week treatment. Asterisks indicate the significant difference (*p* < 0.05) in body gain weight between HGUE and CTL groups in the same treatment week. Each data point represents the mean ± SEM.

**Figure 6:**
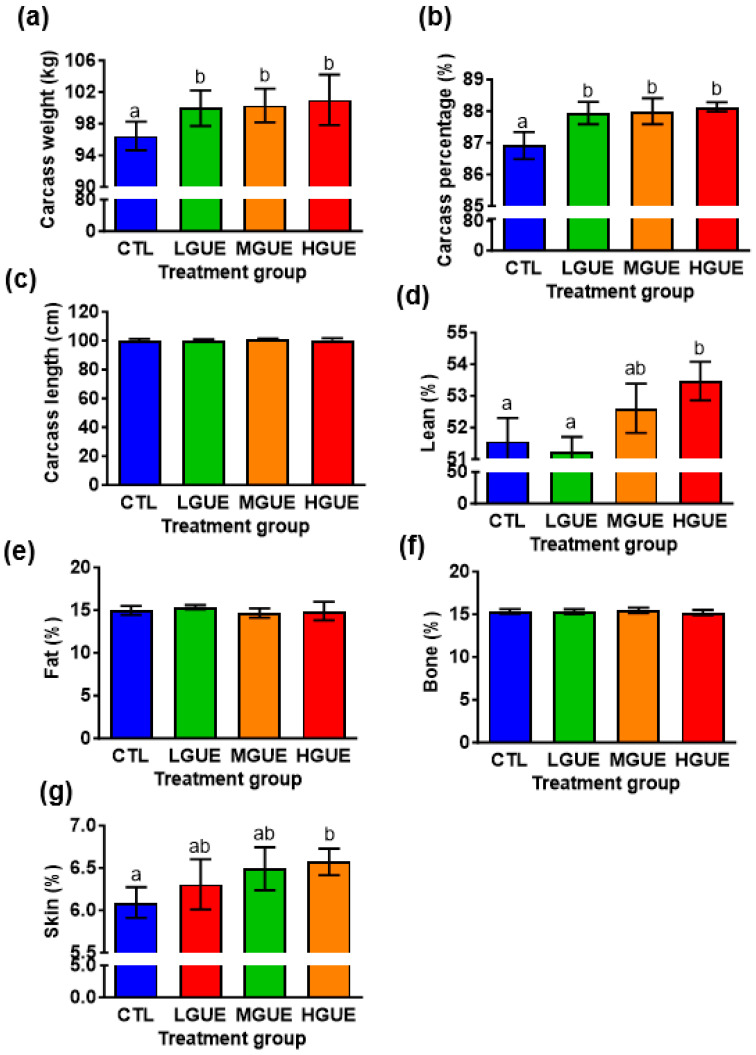
The effect of GUE as a feed additive on carcass characteristics of pigs. Four groups of weaned pigs were fed standard control diet (CTL), or standard diet containing low GUE concentration (LGUE), medium GUE concentration (MGUE), or high GUE concentration (HGUE) for 18 weeks. Carcass weight (**a**), carcass percentage (**b**), carcass length (**c**), as well as the percentage of lean (**d**), fat (**e**), bone (**f**) and skin (**g**) of six representative pigs from each groups were measured and calculated at the end of 18 weeks. Each data point represents the mean ± SEM. Different letters denote statistical significance (*p* < 0.05) among groups.

The effect of GUE as feed additives in growth-finishing pigs was investigated (Figure 5a). The final body weight (BW) showed a significant increase (*p* < 0.05) in the GUE-treated group. In addition, the BW gains in the GUE-treated groups were significantly higher (*p* < 0.05) than that in the control group (Figure 5b,c). There was a significant increase (*p* < 0.05) in average daily gain (ADG) and decrease in feed conversion rate (FCR) in the GUE-treated groups (Table 1). The carcass weight and carcass percentage in the GUE-treated groups were significantly higher (*p* < 0.05) than those in the control group (Figure 6a,b), while there was no significant difference (*p* > 0.05) in carcass length among groups (Figure 6c). The lean and skin percentages of carcass were significant higher (*p* < 0.05) in the GUE-treated groups than the control group (Figure 6d,g), while there was no significant difference (*p* > 0.05) in fat and bone percentages of carcass among groups (Figure 6e,f).

### 3.4. Effect of GUE as a Feed Additive on Gut Microbiota in Growth-Finishing Pigs

**Figure 7:**
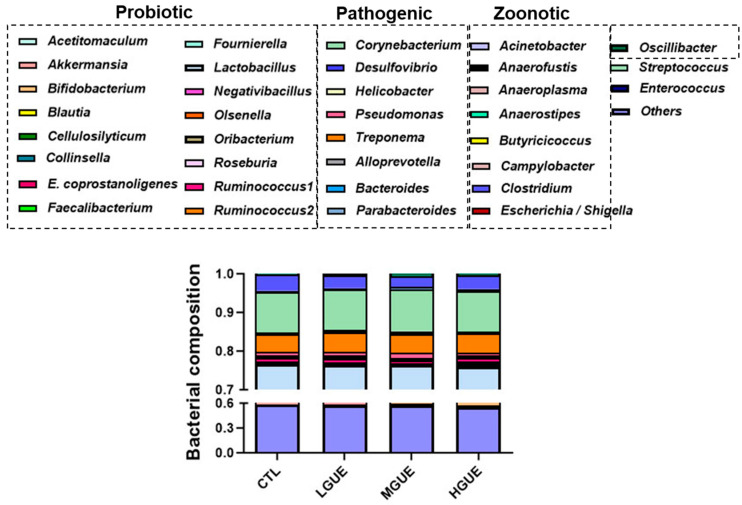
The effect of GUE as a feed additive on gut microbiota in pigs. The bacterial composition at the genus level in the guts of three representative pigs fed control diet (CTL) or the diet containing low GUE concentration (LGUE), medium GUE concentration (MGUE), and high GUE concentration (HGUE) for 18 weeks were pyrosequenced, and the data were analyzed at the end of the 18 weeks.

There have been report indicating a correlation between gut microbiota and growth performance in pigs [42], therefore, the correlation between microbiota and growth performance in pigs fed with GUE was investigated. The results from bacterial composition in pig gut (Figure 7) showed the pathogenic and zoonotic bacteria genera did not differ significantly (*p* > 0.05) in the guts of pigs fed standard diet with or without GUE. Thus, GUE may not increase growth performance through gut microbiota regulation.

## 4. Discussion

The results from several studies indicated that the plant-based or phytogenic feed additives enhance animal health and growth performances [13,18]. These plant-based or phytogenic feed additives were proposed to exhibit antioxidant and antimicrobial properties [16], and also support gut microbiota and functions to improve animal growth performance [18,43]. However, little is known about whether this action was modulated through myogenesis directly during animal growth. In this study, a systematic MSTN expression screening platform was established to screen myogenesis-related phytogenic using L8 cells. MSTN belongs to the TGF-β superfamily and is a secreted protein that acts as a negative regulator in the process of skeletal muscle development [19,20]. MSTN exhibits a high degree of conservation across mammals, encompassing cattle, sheep, pigs, rabbits, and humans. Loss-of-function mutations in MSTN are associated with increased skeletal muscle mass [21,22,23]. Hence, myostatin plays a pivotal role in regulating myogenesis. Inhibition of MSTN expression may have the potential to improve skeletal muscle growth in animals. Presently, a MSTN inhibitor, the monoclonal antibody known as bimagrumab, binds to and restrains the MSTN activity to result in increased muscle mass and strength [44,45]. Clinical trials demonstrated that bimagrumab effectively enhanced muscle mass and function in humans suffering from muscle-wasting conditions, such as sarcopenia and inclusion body myositis [46]. Additionally, MSTN inhibitors could generate potential side effects, including the increased risk of tumor development; thus, further research is necessary to evaluate the safety issue [47]. 

In the livestock industry, there is a growing demand for feed additives that are safe, convenience, and efficiently improve animal growth and performance. Therefore, plant-based or phytogenic feed additive is a better strategy than monoclonal antibody administration to improve animal growth. To rationalize the use of phytogenics, this established systematic MSTN expression screening platform was used to screen various herbal plant extracts which were not only related to skeletal muscle diseases or their regulation, but were also traditionally used as food ingredients and botanical medicine for humans and animals. The results in this study showed that GUE was the only tested plant extract that could increase cell proliferation (Figure 1c) and inhibit MSTN expression in L8 cells (Figure 2a). We also observed that the luciferase activity decrease (Figure 2b) and the ratio of luciferase activity to cell proliferation were used to evaluate the effect of GUE on MSTN expression in L8 cells (Figure 2c). Based on previous reports, MSTN inhibited myogenesis through inhibition of myoblast proliferation [48,49]. 

According to GUE suppressed MSTN expression, and directly promoted L8 myoblast proliferation. These results suggested that GUE has potential as a feed additive and can contain active comportments to promote myogenesis. The process-scale liquid column chromatography was then used to enrich the biological activity of the GUE and identify possible activity components. Using MSTN activity in L8 MSTN-Luc cells as the evaluation method, the efficacy and potency for suppressing MSTN activity in all three phytocompounds were investigated (Figure 3 and Figure 4). These three active compounds, gancaonin G, cudraflavone-C, or licoisoflavone A, suppressed MSTN activity and increased cell proliferation. However, GUE of G9 fraction was more efficacy than these three active compounds (Figure 4c) due to the combination effect of these compounds.

To investigate the potential of GUE as a feed additive. The growth-finishing pigs were fed diets containing different GUE concentration. The body weight and weight gain percentage were significantly increased in GUE-treated groups (Figure 5b,c). As GUE showed myogenesis effect in growth-finishing pigs (Figure 5), the increased body weight gain in pig fed GUE may partially be due to the increase of lean percentage (Figure 6d). The concentration-dependent increase in lean percentage was observed in the GUE-treated groups, thus, higher concentrations of GUE led to greater improvements in lean percentage. There was no significant different in ADFI and TFI with or without GUE treatment (Table 1). There were significant differences in ADG and FCR between control and GUE treatment groups. By modulating MSTN expression in skeletal muscles, GUE may prompt animal growth by increasing body weight and lean content. Consequently, this observation suggests that GUE potentially serve as a favorable feed additive for boosting animal growth under current animal production system.

Akin to most clinical medicine, medical herbs, phytogenic, and phytocompounds have been used in animals through multiple mechanisms, including regulation of cell development, metabolism of nutrients, bacterial composition of gut microbiota, etc. Other studies have also reported that *Glycyrrhiza uralensis*-related products used as feed additives inhibit the relative amount of *Enterobacter*, *Enterococcus*, *Clostridium* and *Bacteroides* [50,51] and increase the relative abundance of *Lactobacillus* [52] at genus levels in weaned piglets. In this study, we analyzed gut microbiota to evaluate the relative amount of probiotic as well as pathogenic and zoonotic bacteria genera in growth-finishing pigs, indicated that GUE did not alter the bacterial composition gut microbiota in growth-finishing pigs (Figure 7). Pigs at the growth-finishing stage display heightened maturity in contrast to their counterparts at the weaned stage. Consequently, the gut microbiota of growth-finishing pigs may exhibit greater stability than that of weaned piglets. Conversely, due to the brief 4 to 5-week span during the weaned piglet stage, the impact of feed additives on gut microbiota during this stage could be relatively effective. In our research, animals fed GUE as a feed additive for 18 weeks did not show significant influence on the gut microbiota of growth-finishing pigs. In this study, the observed GUE-enhanced growth performance may not be due to gut microbiota regulation.

These data suggest a novel application for *G. uralensis* as a feed additive in growth performance enhancement through modulation of MSTN expression. In summary, the edible plant, *G. uralensis*, is recognized as safe for ethnomedicinal or culinary use worldwide [53,54], and the findings from this study along with the published literature confirm the beneficial function of GUE on animal growth.

## 5. Conclusions

The results from the established systematic rodent myoblast screening platform demonstrated the effectiveness in identifying phytogenic compounds by evaluating myostatin promoter activity. Using this screening platform, the GUE was identified and found to significantly increase body weight, carcass weight, and lean content in pigs. In conclusion, this rodent myoblast screening platform successfully identified a myogenesis-promoting phytogenic, GUE, and subsequent experiment showed that the active fraction in GUE inhibited MSTN expression. These findings suggest a novel role for GUE in enhancing growth performance in economic animals by modulating MSTN expression. Furthermore, the established screening platform in this study exhibits the great potential for evaluating a wide variety of phytogenics related to myogenesis.

## Figures and Tables

**Table 1:** Effect of GUE as a feed additive on the parameter of growth performance in pigs.

Treatment group	CTL	LGUE	MGUE	HGUE
Initial body weight (kg)	11.71 ± 1.22	12.62 ± 1.62	12.89 ± 1.18	11.40 ± 1.18
Final body weight (kg)	110.38 ± 6.29 ^a^	118.68 ± 8.12 ^b^	119.48 ± 6.54 ^b^	118.17 ± 10.91 ^b^
Body weight gain (kg)	98.67 ± 7.38 ^a^	106.06 ± 7.48 ^b^	106.59 ± 6.43 ^b^	106.77 ± 9.83 ^b^
ADG (kg/day)	0.78 ± 0.06 ^a^	0.84 ± 0.06 ^b^	0.85 ± 0.05 ^b^	0.85 ± 0.08 ^b^
ADFI (kg/day)	2.00 ± 0.57	2.00 ± 0.42	2.00 ± 0.75	1.98 ± 0.82
TFI (kg)	257.31 ± 7.91	256.92 ± 8.32	257.17 ± 6.11	255.16 ± 5.43
FCR	2.61 ^a^	2.42 ^b^	2.41 ^b^	2.39 ^b^

Four groups of weaned pigs were fed standard control diet (CTL), or standard diet containing low GUE concentration (LGUE), medium GUE concentration (MGUE), or high GUE concentration (HGUE) for 18 weeks. ADG: average daily gain, ADFI: average daily feed intake, TFI: total feed intake, and FCR: feed conversion rate. Each data point represents the mean ± SEM. Different letters (^a^, ^b^) denote statistical significance (*p* < 0.05) among groups.

## Data Availability

The raw data supporting the conclusions of this manuscript are included in this manuscript and Supplementary Information.

## Supplementary Materials

The following supporting information can be downloaded at: https://www.mdpi.com/article/10.3390/bioengineering10101113/s1, Figure S1: Purification and identification of cudraflavone-C from fraction (G9) in *G. uralensis* extract. Figure S2: Myostatin promoter-reporter plasmid.; Figure S3: The luciferase activity assay on dexamethasone and various herb extract-treated L8 MSTN-Luc cells.
