# Supplementary material for: Systematic Myostatin Expression Screening Platform for Identification and Evaluation of Myogenesis-Related Phytogenic in Pigs
Source: Bioengineering (Basel). 2023 Sep 22;10(10):1113. doi: 10.3390/bioengineering10101113 (PMC10604025; doi:10.3390/bioengineering10101113)
Supplement: Supplementary file 1 [file bioengineering-10-01113-s001.zip › bioengineering-2597443-supplementary.pdf]

bioengineering

Supplementary Materials

Systematic Myostatin Expression Screening Platform for Identification and Evaluation of Myogenesis-Related Phytogenic in Pigs

Bor-Rung Ou [1,†], Ming-Hua Hsu [2,†], Ling-Ya Haung [1], Chuan-Ju Lin [3], Li-Li Kuo [3], Yu-Ting Tsai [1], Yu-Chia Chang [3], Wen-Yuh Lin [1], Tsung-Chien Huang [1], Yun-Chu Wu [1], Jan-Ying Yeh [4,*] and Yu-Chuan Liang [3,5,*]

1 Department of Animal Science and Biotechnology, Tunghai University, Taichung 407, Taiwan; brou@thu.edu.tw (B.-R.O.); niyamurry@gmail.com (L.-Y.H.); edhuang68@gmail.com (T.-C.H.)
2 Department of Chemistry, National Changhua University of Education, Changhua 500, Taiwan; minghuahsu@cc.ncue.edu.tw
3 Agricultural Biotechnology Research Center, Academia Sinica, Taipei 115, Taiwan; myd208@gate.sinica.edu.tw (C.-J.L.)
4 Department of Food Nutrition and Health Biotechnology, Asia University, Taichung 413, Taiwan
5 College of Agriculture and Health, Tunghai University, Taichung 407, Taiwan
* Correspondence: jyyeh@asia.edu.tw (J.-Y.Y.); ycliang@gate.sinica.edu.tw (Y.-C.L.)
† These authors contributed equally to this work.

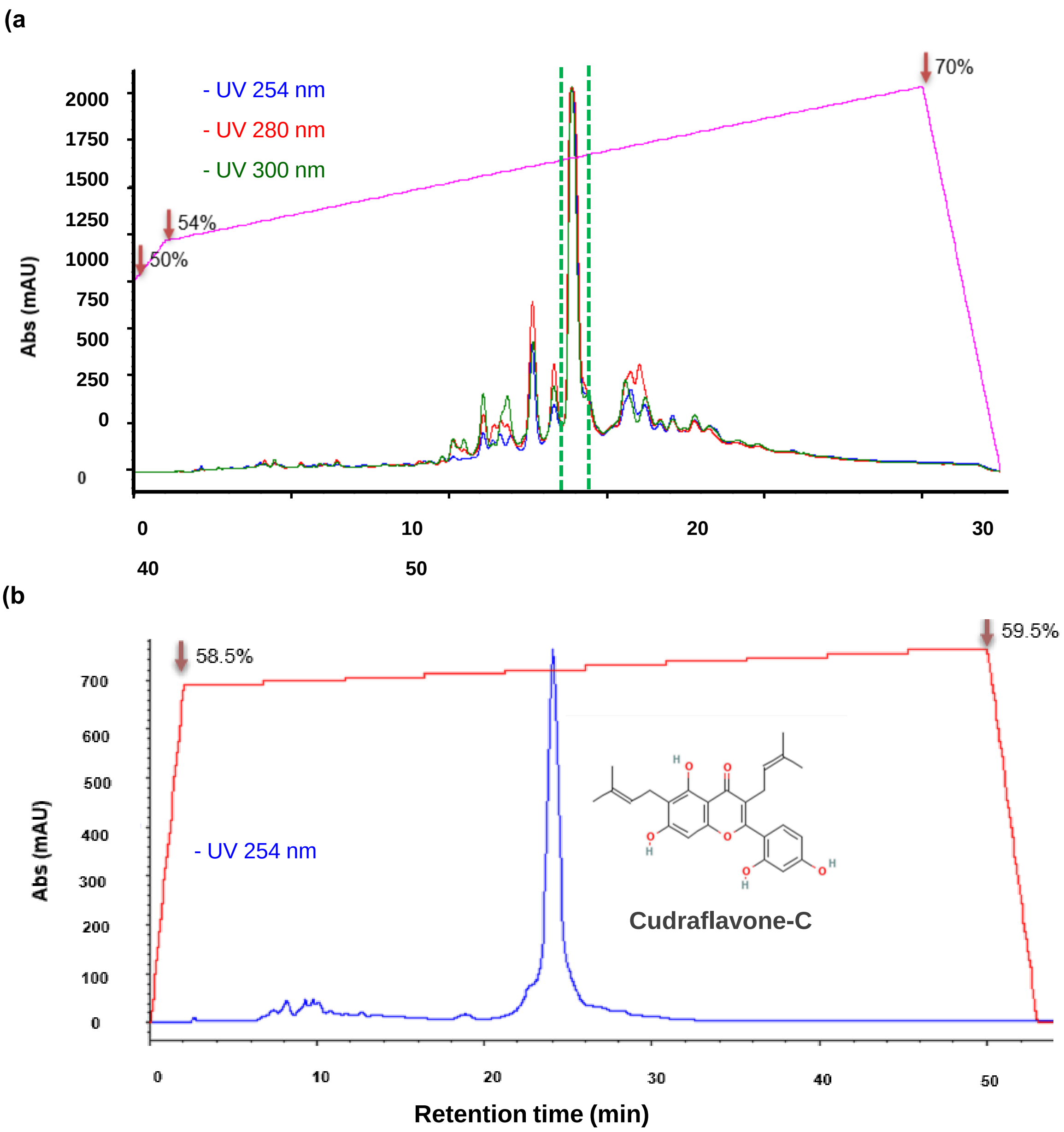

Figure S1. Purification and identification of cudraflavone-C from fraction (G9) in *G. uralensis* extract. The cudraflavone-C was separated from G9 fraction by HPLC between 27 to 29 min of retention time (**a**). Chromatographic profiles of cudraflavone-C was analyzed by LC-MS/MS (**b**).

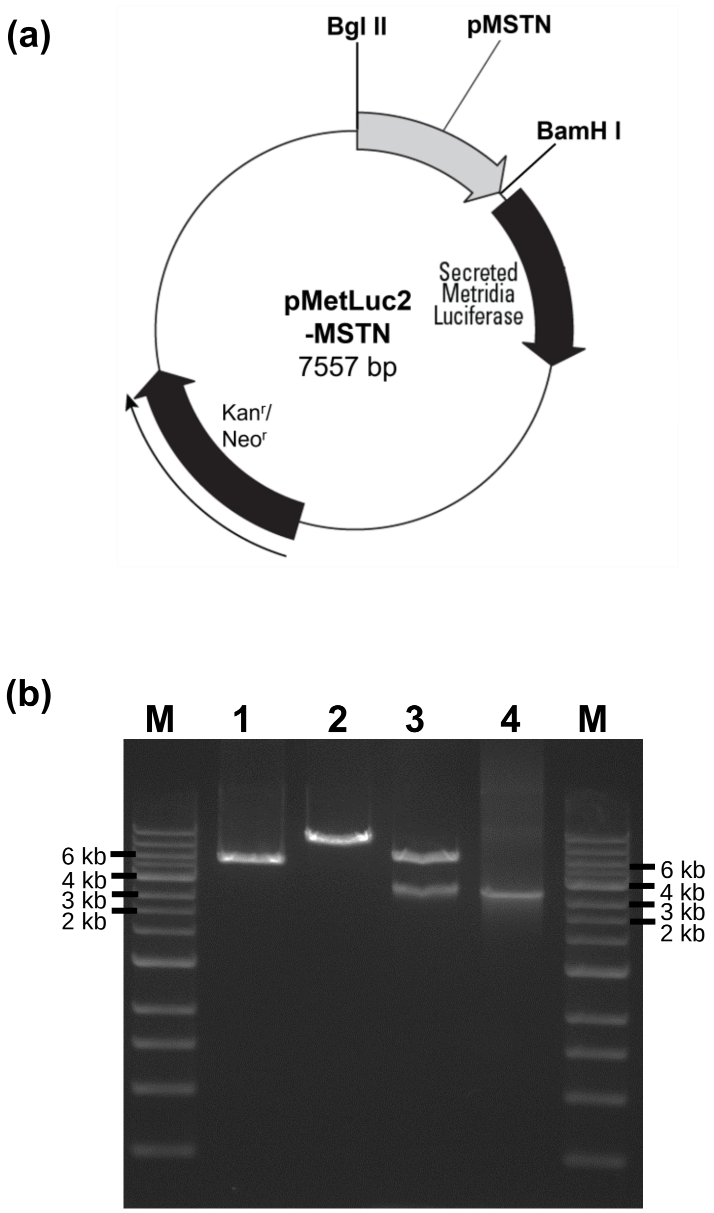

Figure S2. Myostatin promoter-reporter plasmid. The 5′-flanking region (2.46 kb) upstream of the translation start site of the MSTN gene (GenBank accession no. AY204900) was inserted into pMetLuc2 plasmid (a), and confirmed by restriction enzyme digestion (b). Lane 1: pMetLuc2 plasmid after *Bgl*II digestion. Lane 2: pMetLuc2-MSTN plasmid after *Bgl*II digestion. Lane 3: pMetLuc2-MSTN plasmid after *Bgl*II and *Bam*H1 digestions. Lane 4: PCR amplification product of the 5′-flanking region (2.46 kb) upstream of the translation start site of MSTN gene. Lane M: 1-kb DNA ladder.

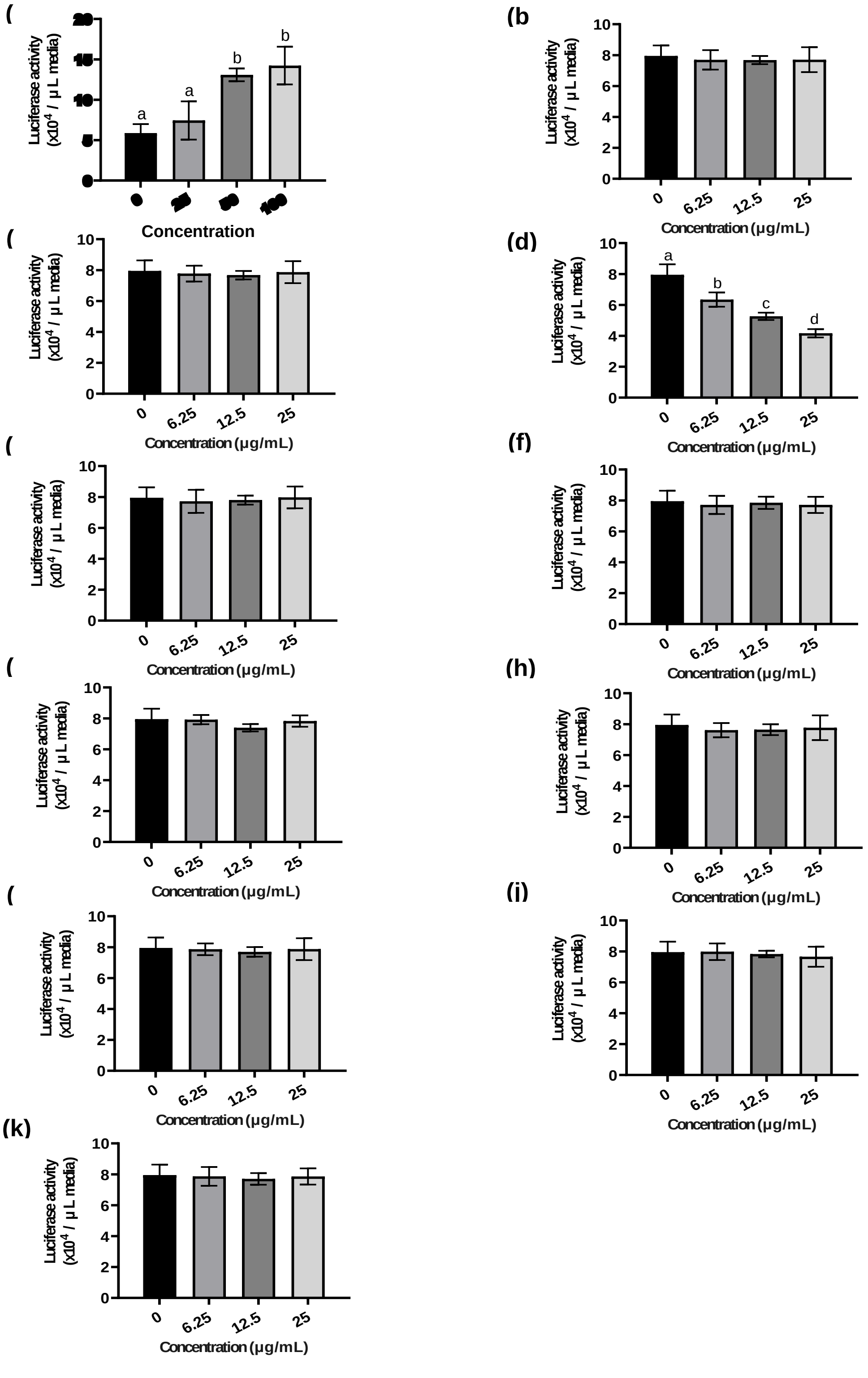

Figure S3. The luciferase activity assay on dexamethasone and various herb extracts treated L8 MSTN-Luc cells. L8 MSTN-Luc cells were treated with dexamethasone (**a**) or various herbal extracts, *Andrographis paniculate* (**b**), *Centella asiatica* (**c**) , *Glycyrrhiza uralensis* (**d**), *Gynostemma pentaphyllum* (**e**), *Platycodon grandifloras* (**f**), *Polygonum chinense* (**g**), *Portulaca oleracea* (**h**), *Saururus chinensis* (**i**), *Smilax china* (**j**) and *Taraxacum campylodes* (k). L8 MSTN-Luc cells luciferase activity was measured after 24h treatment. Each data point represents the mean ± SEM. Different letters (a–d) denote significant differences ($p < 0.05$) amount treatments.
